# Antenatal macrosomia prediction using sonographic fetal abdominal circumference in South Tunisia

**DOI:** 10.11604/pamj.2013.14.111.1979

**Published:** 2013-03-21

**Authors:** Kais Chaabane, Khaled Trigui, Doulira Louati, Sahbi Kebaili, Hichem Gassara, Abdallah Dammak, Habib Amouri, Mohamed Guermazi

**Affiliations:** 1 Obstetrics and Gynecology Department, Hédi Chaker Academic Hospital- Sfax-Tunisia

**Keywords:** Fetal abdominal circumference, macrosomia, Ultrasonography

## Abstract

**Introduction:**

Identifying newborns who weight 4000 g or more is important because birth of macrosomic fetuses is associated with adverse peripartum outcomes. Ultrasound is widely used for this purpose Our objective was to evaluate the diagnostic value of sonographic measurement of fetal abdominal circumference (AC) over 350 mm for the prediction of fetal macrosomia and shoulder dystocia, to specify factors that could generate errors in its measure.

**Methods:**

A retrospective clinical trial was conducted at the Department of Obstetrics and Gynecology, Hédi Chaker Hospital, Sfax, Tunisia. The study consisted of comparing two groups of singleton newborns: the first group (n=465) includes macrosomic babies and the second group (n=465) includes the non macrosomic ones. All women underwent sonographic measurements of the fetal abdominal circumference (AC) within 72 hours before delivery. The AC values were correlated to actual fetal birth weight. The cut-off value of AC for predicting of fetal macrosomia was analyzed.

**Results:**

A cut-off value of abdominal circumference ≥350 mm, in predicting of fetal macrosomia., had a sensitivity, specificity, accuracy, positive predictive value, and negative predictive value: 78.7%, 76.8%, 77%, 92.6%, and 49.2%, respectively. In macrosomic group obesity was significantly more frequent when AC≥350mm.

**Conclusion:**

The fetal AC measurement was useful in predicting of fetal macrosomia. An AC measurement AC≥350mm could help to suspect shoulder dystocia.

## Introduction

Fetal macrosomia defined as infant birth weight ≥4000g, is associated with many adverse outcomes, which can be the cause of high rate perinatal maternal and fetal morbidity and mortality. Accurate methods for prenatal prediction of macrosomia would therefore be very useful for planning labor and delivery strategies and consequently prevent from its complications. Ultrasonographic assessment of fetal growth to estimate fetal weight is widely used in obstetrics. Several criteria and models to calculate them are proposed. The purpose of this study was to evaluate the diagnostic value of ultrasonographic measurement of fetal abdominal circumference over 350 mm for the prediction of fetal macrosomia, to determine factors associated with the failure to detect this threshold in some macrosomic infants, and to assess its value in predicting shoulder dystocia.

## Methods

A retrospective controlled case study was conducted between January 2007 and December 2008 in the Department of Obstetrics and Gynecology of Sfax Tunsia. During this period, we registered a total of 18289 deliveries, including 1283 macrosomic infants (7%).

Stastical comparaison was performed between a group of women delivering liveborn neonates between 37 and 42 weeks weighing more than 4000g and a group of women with the same inclusion criteria except infant weight which was less than 4000g. For all women the sonographic examination must have been performed within 72 hours before delivery, in order to eliminate the possible effect of the time elapsed between ultrasound abdominal circumference examination and delivery for actual fetal weight. Finally the two groups included 465 women each.

A regression analysis was used to evaluate the best linear relationship between abdominal circumference (AC), biparietal diameter (BPD), femur length (FL) and birth weight (BW). Odds ratios for each criteria, were calculated, and significance was tested with the Chi 2 test.

To evaluate factors associated with the failure of detection of an AC≥350 mm in macrosomic neonates, we compared maternal characteristics in two groups of this population: the group 1 includes macrosomics with an AC over 350mm and group 2 enclosing macrosomics with an AC less than 350mm. Statistical comparison was made by « chi 2 » for the frequencies and « Student » test for the means. Logistic regression analysis was used to determine significant predictors of shoulder dystocia.

## Results

The median of AC was 367.7mmm in the macrosomic group, and 330.4 mm in the non macrosomic. The difference was significant (Table 1). Chi square regression analysis using birth weight as dependant variable and AC, BPD and FL as independent variable showed that AC alone was the best factor predicting birth weight in macrosomic neonates (r=0.8, P<0.001).compared with the BPD (r=0.67, P<0.001) and FL (r=0.7, P<0.001).

**Table 1:** The Abdominal circumference > 350 mm in predicting fetal macrosomia

	Macrosomic	Non macrosomic	P
Median of abdominal circumference	367.7mm	330.4mm	< 0,001
AC<350 mm	21.3%(n=99)	76.5%(n=356)	< 0,001
AC≥350 mm	78.7%(n=366)	23.5%(n=109)	< 0,001
BW	4152g	3360	<0.001

AC: Abdominal circumference; BW: Body weight

A cut-off value AC over 350mm,was found significantly more frequent in macrosomic (78.7% n=366) compared with the non macrosomic (23.5% n=109) (Table 1).The accuracy and predictability of 350 mm AC for detecting macrosomia were sensitivity (78.7%), specificity (76.8%), negative predictive value (49.2%), and positive predictive value (92.6%), (Table 2).

**Table 2:** The diagnostic value of AC>350 mm in predicting fetal macrosomia

Sensitivity	78.7%
Specificity	76.8%
Positive predictive value	92.6%
Negative predictive value	49.2%
Accuracy	77%

In the macrosomic group we noticed that 21.3% (n=99) of this population had AC less than 350 mmm. Analysing factors that could be interfering with the detection of this threshold in macrosomic fetuses, we found that a history of macrosomia was more frequent in group 1 than in group 2 (7.6% versus 4%, p<0.001).Body mass index (BMI) over 30 was more frequent in group 2 (31.3% versus 17.4%, P<0.001).There was no significant difference in maternal age (P=0.2),gestational age (P=0.2), neither in BMI between 25 and 29 (P=0.12). Actual birth weight was statististically not different between both groups (4187g in group 1, 4123g in group 2, p=0.18) (Table 3).

**Table 3:** Maternal characteristics in macrosomic group

	AC≥350mm (n=366)	AC<350mm (n=99)	P
Median of age (years)	30.6	30.2	0.2
Gestational age (weeks)	40 weeks+1 day	40 weeks+4 days	0.2
Parity	2.32	2.64	0.36
Macrosomia history	7.6%(n=28)	4%(n=4)	<0.001
BMI≥30	17.4%(n=64)	31.3%(31)	<0.001
25<BMI<29	34.7%(n=127)	33.3%(n=33)	0.12
BW	4187g	4123g	<0.001

AC: Abdominal circumference BMI: Body mass index BW: Body weight

In macrosomic neonates, shoulder dystocia occurred in 67 cases when AC≥350mm (18.3%) and in 10 cases when AC<350mm (10.1%), P<0.01. In non macrosomic infants, shoulder dystocia was found in 3 cases when AC≥350mm (2.8%) and in 3 cases when AC< 350mm (0.8%), P<0.01 (Table 4).

**Table 4:** Incidence of shoulder dystocia according to the threshold of 350mm

	AC≥350mm	AC<350mm	P
Macrosomic	18.3%(n=67)	10.1%(n=10)	<0.01
Non macrosomic	2.8%(n=3)	0.8%(n=3)	<0.01

AC: Abdominal circumference

Logistic regression, with shoulder dystocia as independent variable and AC≥350 mm as dependant variable showed than AC over a cut off value of 350mm alone was associated with shoulder dystocia (OR=2.85, 95%confidence interval 1.4, 7.4, P<0.01).

## Discussion

Macrosomia is a cause of the worst of obstetric emergencies such as shoulder dystocia, birth asphyxia, and postpartum hemorrhage [1, 2]. About half of shoulder dystocia happen to macrosomic infants, yet frequency of macrosomia is less than 10%. Shoulder dystocia cannot always be predicted accurately. However, predicting macrosomia can help to identify the population at the highest risk for shoulder dystocia. Several studies of sonographic measurement for predicting of fetal macrosomia were established [3–5].

Campbell and Wilkin [6] first emphasized the importance of ultrasound AC measurements in determining fetal size in 1975. Smith et al compared the accuracy of two sonographic fetal weight-estimation models and AC as a single measure for the prediction of fetal macrosomia and found that models based on three biometric indices including AC, BPD, and FL appeared to be less accurate for the diagnosis of fetal macrosomia than models based on AC as a single measure [7].

Several studies have shown that an AC measurement ≥350 mm was the best predicting value of fetal macrosomia [8, 9].This conclusion was matching with our results with an accuracy of 77%.

Comparing our results with previous studies, we found that the negative predictive value was lower (49.2%). So we analysed factors that could be associated with the failure of detection an AC≥350mm in macrosomic infants, we found that obesity was significantly higher when AC<350mm.This may be explained by the fact that ultrasound performance could be minimized by maternal adiposity [10].

Shoulder dystocia remains a serious obstetric emergency and although not always predictable, it is clear that large fetuses are at greatest risk for it. Sonographic detection of this complication was a subject of high interest for clinicians. Jazayeri and al found that all infants presenting shoulder dystocia had an AC more than 350mm, regardless the actual birth weight [11]. Inour study we found similar results.

We emphasize that the population used in this study was larger than previous studies [4, 5, 11]. Most of other researches included about one hundred samples. In this study, we included 465 samples hoping to decrease the bias of sample distribution.

The accuracy of our retrospective study in this population of pregnant women needs to be tested in other populations to confirm its applicability. Because not all patients at risk for shoulder dystocia can be identified, all persons delivering infants must be trained in its appropriate management.

## Conclusion

Abdominal circumference measurements were useful in screening for suspected macrosomia, An AC measurement of 350 mm or more could help to suspect shoulder dystocia, and therefore motivate obstetricians to be more vigilant during the delivery.
